# Cultivating a Patient-Centered Environment (CAPE): Renovations in Long-Term Chronic Care

**DOI:** 10.1093/geroni/igab046.3190

**Published:** 2021-12-17

**Authors:** Elizabeth Howard, Tammy Retalic

**Affiliations:** 1 Boston College, Dover, Massachusetts, United States; 2 Hebrew SeniorLife, Boston, Massachusetts, United States

## Abstract

Achieving institutional goal of full, person-centered care was encumbered by an outdated structural “hospital model” at one long-term care facility that undertook building renovations, transforming long hallways into “neighborhood” of compact households. Quality of Life Survey and Long-term Care Minimum Data Set generated data at baseline and 1-year follow-up, comparing renovated(RU) and non-renovated unit(NRU) residents (n=36) to evaluate achievement of person-centered care. RU residents indicating they could “eat when I want” increased 75% to 81% at follow-up and decreased 17% for NRU residents. Sixty-seven percent of RU residents reported bathing “when they want” in contrast to 40% of NRU residents. Most RU residents agreed, “staff act on my suggestions.”More RU residents (68% vs 53%) agreed: “I spend time with other like-minded residents” and more RU residents (86% vs 43%) reported opportunity to explore new skills, interests. RU residents more often reported (50% vs 37%) “people ask for my help or advice.” Similar differences were observed with “it is easy to make friends here,” 67% RU residents responding affirmatively. RU residents reporting “feeling down” improved, moving from 46% to 50% disagreeing with this item with while increased number of NRU residents (18% to 22%) reported “feeling down” at follow-up.Improvement with independent performance of bed mobility, transfer, walking, and dressing among RU residents was observed while NRU residents had decreased percentages of independence. Evaluation of resident outcomes demonstrated improvement with personal choice, activities, personal relationships, functional independence and mood. Physical unit renovations appear to enhance implementation of person-centered care model.

